# Incidence of Vancomycin Resistant Phenotype of the Methicillin Resistant *Staphylococcus aureus* Isolated from a Tertiary Care Hospital in Lahore

**DOI:** 10.3390/antibiotics9010003

**Published:** 2019-12-18

**Authors:** Aqib Saeed, Fatima Ahsan, Muhammad Nawaz, Khadeja Iqbal, Kashif Ur Rehman, Tayyaba Ijaz

**Affiliations:** 1 Department of Microbiology, University of Veterinary and Animal Sciences, Lahore 54000, Pakistan; aqibs114@gmail.com (A.S.); fatima.ahsan@uvas.edu.pk (F.A.); 2 Department of Microbiology, Central Diagnostic Lab, King Edward Medical University/Mayo Hospital Lahore, Lahore 54000, Pakistan; drkhadeja@yahoo.com; 3 Department of Pathology, Central Diagnostic Lab, King Edward Medical University/Mayo Hospital Lahore, Lahore 54000, Pakistan; rehmankashif1004@gmail.com (K.U.R.); tayyabalhr@yahoo.com (T.I.)

**Keywords:** *S. aureus*, MRSA, VRSA, molecular detection

## Abstract

*Staphylococcus aureus* (*S. aureus*)-associated infections are one of the major threats to public health. The aim of the present study was to determine the antibiotic resistance pattern as well as the genetic characterization of methicillin and vancomycin resistant *S. aureus* (VRSA) isolated from a tertiary care hospital in Lahore. The *S. aureus* isolates were isolated from different clinical samples, identified by biochemical testing, and subjected to antibiotic susceptibility testing via the disc diffusion method or broth microdilution method. The methicillin resistance gene (*mec*A) and vancomycin resistance gene (*van*A) were amplified by the polymerase chain reaction. The *S. aureus* isolates showed high incidences of resistance against methicillin (76%) and moderate incidences of resistance to vancomycin (14%). Isolates were also resistant to several other drugs, such as cefoxitin (76%), ertapenem (83%), ampicillin (81%), tobramycin (78%), moxifloxacin (76%), and tetracycline (74%). An encouraging finding was that 98% of isolates were susceptible to tigecycline, indicating its possible role in the treatment of methicillin-resistant *Staphylococcus aureus* (MRSA) and VRSA, as well as the multi-drug resistant *S. aureus*. The *mec*A gene was detected in 33.3% of tested isolates (10/30), while the *van*A gene was also detected in 30% (9/30) of the tested isolates. In conclusion, the frequent presence of methicillin and vancomycin resistance in *S. aureus* appraises the cautious use of these antibiotics in clinical practices. Furthermore, it is suggested that there should be continuous monitoring of tigecycline treatments in clinical setups in order to delay the development of resistance against it.

## 1. Introduction

*Staphylococcus aureus* (*S. aureus*) infection is one of the most common nosocomial and community-acquired infections in humans and animals [1,2]. Almost 30% of the human population is asymptomatically colonized with commensal *S. aureus* [3,4]. The *S. aureus* is capable of causing a variety of diseases including pneumonia, mastitis, meningitis, wound infections, sepsis, abscess formation, osteomyelitis, endocarditis, food poisoning, and toxic shock syndrome (TSST) [5]. Transfer of antibiotic resistance genes is common in *Staphylococcal* species [6]. Resistance against methicillin, lincosamides, macrolides, aminoglycosides, and a combination of these antibiotics have been frequently reported in staphylococci [7]. Methicillin resistance is generally caused by *mec*A gene that encodes polypeptide PBP2a protein [8]. Within the past few decades, there has been an alarming increase in prevalence of antibiotic resistance in pathogens as well as in commensals [2]. Glycopeptides, especially vancomycin, are generally used against methicillin-resistant *Staphylococcus aureus* (MRSA) since the last few decades of 20th century [9]. Irrational use of antibiotics has led to the emergence of vancomycin resistance in *S. aureus* strains [10]. Vancomycin-resistant *Staphylococcus aureus* (VRSA) strains may contain different vancomycin resistance genes, including *van*A and *van*B gene [11]. MRSA infections has led to the increased use of glycopeptides, especially vancomycin, and frequent emergence of VRSA phenotype among MRSA [12].

The MRSA have been frequently reported from *S. aureus* isolated from different clinical setups [13]. The current study was planned to determine the antibiotic resistance pattern of clinical isolates of *S. aureus,* and detection of resistance genes (i.e., *mec*A and *van*A) which cause resistance against methicillin and vancomycin, respectively. This study was also an attempt to explore an effective antibiotic against MRSA and VRSA.

## 2. Results

The overall result of the current study revealed that clinical isolates of *S. aureus* (*n* = 100) had a high incidence of resistance against methicillin (76%), ampicillin (81%), tobramycin (78%), tetracycline (74%), aztreonam (85%), cefoxitin (76%), and ertapenem (83%), and a moderate occurrence of resistance against vancomycin (14%). The isolates that were resistant to cefoxitin were declared as MRSA [14]. Most of the *S. aureus* isolates (98%) were susceptible to tigecycline (Table 1).

The highest occurrence of MRSA was identified from pus samples (29/36, 80.55%) followed by sputum (19/24, 79.16%), blood (19/25, 76%), and other body fluids (9/15, 60%). Specimen wise distribution also showed that frequency of occurrence of VRSA among MRSA was the highest in sputum samples (13/19, 68.42%) followed by blood (12/19, 63.16%), body fluids (5/9, 55.56%), and pus (14/29, 48.28%) (Table 2). The Chi-square test was applied to check the significance of association of the type of sample to the incidence of MRSA. We could not find any significant association between these variables, and it appears that occurrence of the MRSA is independent of the site of infection. The background detail on patient history was also collected at the time of sample collection from the patients (Table 3). This information shows a general lack of knowledge regarding the use of antibiotics and common practice of leaving the medication half-way without completing the prescribed dosage of antibiotic treatment (Table 3). This information provides an insight into the background factors that contribute to the emergence of antibiotic resistance strains in population.

### 2.1. Antibiotic Susceptibility Pattern of MRSA

Among 100 isolates of *S. aureus*, 76 were resistant to methicillin, all of which (100%) were also resistant to ampicillin and ertapenem (Table 4). MRSA isolates were also found to be resistant to tobramycin (72, 94.7%), tetracycline (70, 92.1%), cefoxitin (76, 100%), and moxifloxacin (68, 89.4%). MRSA isolates also showed co-resistance with vancomycin (14, 18.42%). Out of 76 MRSA isolates, only one (1.3%) isolate was resistant to tigecycline.

### 2.2. Antibiotic Susceptibility Pattern of VRSA/VISA Isolated from MRSA

The antibiotic susceptibility pattern of the 44 VRSA/VISA isolates is presented in Table 5. The vancomycin-resistant phenotype was confirmed by determining the minimum inhibitory concentration (MIC) of isolates against vancomycin via the broth micro-dilution method, as described by the European Committee for Antimicrobial Susceptibility Testing (EUCAST) of the European Society of Clinical Microbiology and Infectious Diseases (ESCMID) and the Clinical Laboratory Standards Institute (CLSI) [15,16]. The MICs of vancomycin for 44 reported VRSA/VISA showed that 14 isolates had MICs >16 µg/mL while the other 30 isolates had MICs in the range of 2–4 µg/mL, revealing that these were vancomycin-intermediate *S. aureus* (VISA). The data also showed that most of the VRSA/VISA isolates were also resistant to ampicillin (44/44, 100%), cefoxitin (44/44, 100%), tobramycin (43/44), tetracycline (42/44), and moxifloxacin (41/44). Only one VRSA/VISA isolate was found resistant to tigecycline (2.2%) (see Table 5).

### 2.3. Amplification and Partial Sequencing of mecA and vanA

**Figure 1:**
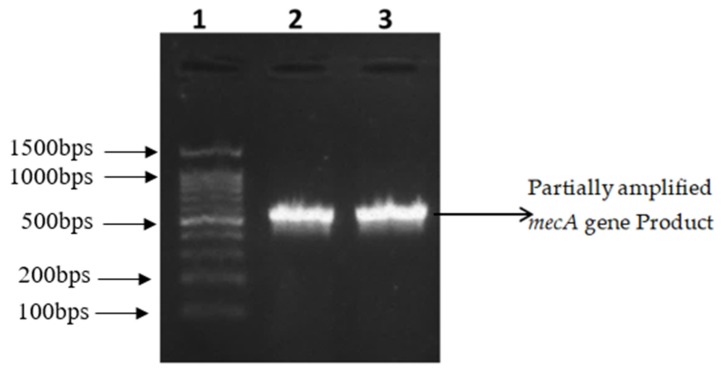
Partially amplified *mecA* gene Product (583 bps). Lane 1: 100 bp DNA ladder RTU, Cat # DM001-R500. Lanes 2, 3: Representative isolates with amplified gene product.

The resistance genes *mec*A and *van*A were amplified by polymerase chain reaction using gene-specific primers as described in Table 6. Thirty isolates that were phenotypically resistant to methicillin and vancomycin were selected for the identification of resistant genes. Methicillin resistant gene (*mec*A) partial region was successfully amplified from 10 (33.33%) out of 30 MRSA isolates (Figure 1).

**Figure 2:**
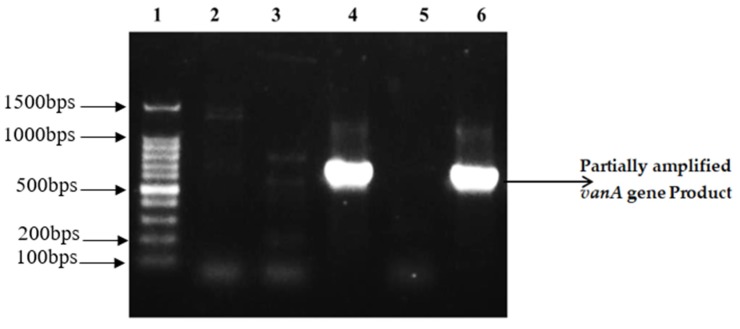
Partially amplified *van*A gene Product (713 bps). Lane 1: 100 bp DNA ladder RTU, Cat # DM001-R500. Lane 2, 3 & 5: Representative isolates where no amplification was observed. Lane 4 & 6: Representative isolates with amplified gene product.

Out of 30 isolates, the vancomycin resistant gene *van*A was amplified from nine (30%) isolates (Figure 2). The target sequences of *mec*A and *van*A genes were sequenced to confirm the identity of the amplicons by Sanger’s dideoxy chain termination sequencing method in order to exclude any non-specific amplification.

## 3. Discussion

Infections due to MRSA and VRSA/VISA are associated with high morbidity and mortality. Within the past few years, the prevalence of MRSA has reached a disturbing level, not only in developed countries but also in Pakistan [7,17]. Rapid emergence of resistance in bacteria is associated with the overuse and misuse of antibiotics in clinical and veterinary set-ups [18,19]. The present study reported the isolation of MRSA (76) and VRSA/VISA (44) phenotypes from 100 clinical samples which is consistent with previous studies from other South Asian countries. A study conducted in Bangladesh also reported that 72.4% of clinical isolates of *S. aureus* were resistant to methicillin while 37.9% were resistant to vancomycin [20]. A study from the Indian state of Hyderabad reported that a large number of *S. aureus* strains (79.6%) isolated from clinical specimens were MRSA [21]. A similar study from Uttar Pradesh reported 54.85% in MRSA phenotypes among isolated *S. aureus* [22]. The Pakistani population is also facing similar challenges as their neighboring countries since two independent studies from Rawalpindi and Rahimyar Yaar Khan have also found 60.4% and 66.7% incidence of MRSA [23,24].

In the present study, 44 out of 76 MRSA isolates were also phenotypically resistant to vancomycin, which is quite different from a previous study from Bangladesh which reported that none of the MRSA isolates were resistant to vancomycin [25]. Vancomycin resistance in *S. aureus* (2.5%) has been determined and reported from clinical set ups from Pakistan previously as well [26]. A study from Kasur, Punjab reported that 84.6% of *S. aureus* isolated from bovine sub-clinical mastitis were resistant to vancomycin which indicate that vancomycin resistance is on the rise in veterinary set ups as well [27].

A specimen wise distribution of the VRSA/VISA and MRSA population was also statistically analyzed by Chi-square test for the significance of association. It shows a *p* value greater than 0.05, which means there is no significant association between the incidence of MRSA or VRSA to the type of specimen (Table 2). The number of VRSA/VISA, isolated here, indicates an alarming condition. The *van*A gene provides a high level of vancomycin resistance (MIC 512–1024 µg/mL) and is frequently found in enterococci. This can be transferred via plasmid from enterococci to a resident MRSA strain resulting in the development of VRSA/VISA [7]. Out of the 30 MRSA + VRSA/VISA isolates in the present study, only 10 isolates were positive for *mec*A gene and 14 were positive for *van*A gene. Occurrence of *mec*A negative methicillin resistant phenotypes can be explained by limitations of testing methods as well as the possibility of development of other mechanism of methicillin resistance. Absence of *mec*A from MRSA has been reported previously as well [28]. Similarly, *van*A negative VRSA/VISA isolates shows that other vancomycin resistant determinants (*van*B) or mechanisms may also be prevalent in *S. aureus* which should be explored and monitored closely in future [29]. The major reasons associated with the rise of resistance phenotype are the lack of awareness and the realization of the impact of auto-medication and unnecessary use of the antibiotics in the population. Another factor is lack of proper regulations for the control of the antibiotic sale without prescription. Proper campaigns are required to increase the awareness among the general population about the impact of individual actions contributing to the development of resistance amongst the bacteria. It is also noteworthy that a very common practice that the patients do not follow the course of treatment of antibiotic as per instructions of the medical practitioners. Most of the people leave the consumption antibiotics mid-way as soon as there is some relief from fever and a decline in the apparent symptoms. This attitude is reflected in the information collected at the time of sample collection as shown in Table 3. The overall attitude of the population could only be changed by proper education and taking the measures to increase awareness among the general public. This also necessitates the formulation of strict regulations regarding the use of antibiotics without a prescription. The variation in the observations among different studies is due to nature of different strains at different localities, hospital infection control strategies, and other measures taken to control the use of antibiotics. In the present study, VRSA/VISA showed resistance to a wide range of antimicrobial agents. However, the majority of MRSA and VRSA/VISA were sensitive to tigecycline, indicating a possible role of tigecycline in control and treatment of VRSA/VISA. Efficacy of tigecycline against MRSA and VRSA/VISA infections has been reported previously as well [5]. Linezolid and Quinopristin/Dalfopristin are other alternative treatment choices against MRSA and VRSA/VISA [30].

## 4. Materials and Methods

### 4.1. Isolation and Identification of S. aureus

*S. aureus* isolates were collected from pathology laboratory of a tertiary care hospital in Lahore from December 2017 to April 2018. Data was also collected including patient’s history, age, gender, and type of sample (i.e., blood, pus, sputum, and other body fluids). Isolates were grown on mannitol salt agar at 37 °C. Isolates were confirmed as *S. aureus* by culture, microscopic, and biochemical characters such as Gram staining, oxidase, catalase, coagulase, motility, DNase, haemolysis, and mannitol fermentation tests [12].

### 4.2. Antibiotic Susceptibility Testing

Antibiotic susceptibility testing was performed using disc diffusion method [31]. *S. aureus* ATCC 29,213 was used as reference strains for quality control in antibiotic susceptibility testing. Briefly, bacterial inoculum (~0.5 McFarland, from each isolate) was swabbed on a separate Muller Hinton agar (HiMedia Laboratories Pvt. Ltd., Mumbai, India) to obtain a lawn of confluent growth. Antibiotic discs used in this study were procured from Oxoid Limited, UK, except methicillin disc which was obtained from Abtek Biological Ltd., Liverpool, UK. The discs of antibiotics including methicillin (5 μg), ampicillin (10 µg), tobramycin (10 µg), tetracycline (30 µg), moxifloxacin (5 µg), cefoxitin (30 µg) and ertapenem (10 µg), and tigecycline (30 µg) were placed on inoculated plate followed by incubation at 35 °C for 16 h. After 24 h, zone of inhibition around antibiotic discs were measured and organisms were designated as resistant, sensitive, or intermediate according to breakpoints provided by Clinical and Laboratory Standard Institute (CLSI), Wayne, PA, USA.

### 4.3. Phenotypic Detection of MRSA

Detection of MRSA was carried out by using cefoxitin disc as recommended in literature [31,32]. All isolates were subjected to cefoxitin disc diffusion test by using 30 µg cefoxitin discs. A 0.5 McFarland bacterial inoculum was prepared and swabbed on the Muller Hinton agar plate to obtain a lawn of confluent growth. The plates were incubated at 35 °C for 16 h and zone diameters were measured, as recommended by CLSI.

### 4.4. MIC (Minimum Inhibitory Concentration) for the Confirmation of VRSA/VISA

For the confirmation of VRSA/VISA, minimum inhibitory concentration was performed by broth micro-dilution method as described by European Committee for Antimicrobial Susceptibility Testing (EUCAST) of the European Society of Clinical Microbiology and Infectious Diseases (ESCMID) and Clinical Laboratory and Standard Institute (CLSI) [15,16]. Briefly described, two fold serial dilutions (0.25 µg/mL–256 µg/mL) were prepared in Muller Hinton Broth by using microtiter plate wells. Double diluted antibiotics (50 µL) were inoculated with *S. aureus* (100 µL). *S. aureus* inoculum (0.5 McFarland) was made by suspending their fresh growth in normal saline. The inoculum is further diluted in Muller Hinton broth in order to obtain a final organism density of 5 × 10^5^ CFU/mL (Range 3–7 × 10^5^ CFU/mL). The microtiter plates were incubated at 37 °C for 24 h. After 24 h, MICs were calculated by taking OD at 630 nm. *S. aureus* ATCC 29,213 was used as reference strains for quality control.

### 4.5. Amplification of Methicillin and Vancomycin Resistance Genes

The total DNAs were extracted by using a commercial Kit GeneAll (South Korea). The extracted DNA was then visualized by agarose gel electrophoresis. The purified DNA was then subjected to PCR based amplification of *mec*A and *van*A gene by using specific gene primers (Table 6) as described previously [33,34]. The primers were synthesized on order by Macrogen, Inc., Seoul, Korea. For *mec*A and *van*A PCR assays, in-house strains (*S. aureus* 113 and *Enterococcus* *faecium* 83, respectively) were used as positive control strains.

PCR reaction (25 µL) contained 12.5 µL of master mix (WizPure PCR^TM^ 2× Master, Wiz bio solutions), 10 p mol of respective primers and 25 ng of DNA. Amplification was carried out in a thermocycler (T100^TM^, Bio-Rad) using the following thermal settings: initial denaturation for 5 min, followed by 40 cycles of denaturation at 94 °C 30 s, annealing at 58 or 57 °C for 1 min for *van*A and *mec*A, respectively, extension at 72 °C for 1 min, and final extension at 72 °C for 5 min. The amplified product was then visualized by agarose gel electrophoresis.

### 4.6. DNA Sequencing for mecA and vanA Gene Detection

In order to rule out non-specific amplification and to confirm that the amplified product contains the target sequence of *mec*A and *van*A genes, these were subjected to DNA Sequencing by Sanger’s dideoxy chain termination reaction method. Gene specific primers were used to sequence the purified amplicon from the above PCR reactions.

## 5. Conclusions

The current study reports a high recovery of MRSA and VRSA/VISA from clinical samples in Lahore. VRSA/VISA is becoming a potential hazard for public health, since vancomycin has been considered as the drug of choice against *S. aureus* in the settings where MRSA are prevalent. The emergence of VRSA/VISA in such case requires the use of alternative antibiotics. Thus, vancomycin in clinical settings should be used cautiously to curtail the appearance and spread of new resistant strains. Fortunately, tigecycline could still be used effectively for the treatment of these infections. Further long term studies are essential to explore the epidemiological patterns and resistance mechanism of VRSA/VISA in different areas of Pakistan.

## Figures and Tables

**Table 1:** Antibiotic sensitivity pattern of *Staphylococcus aureus* (*n* = 100).

Antibiotic	Quantity (µg)	Antibiotic Sensitivity Pattern of *S. aureus* (*n* = 100)		
		Resistant	Intermediate	Sensitive
Methicillin *	5	76	11	13
Ampicillin *	10	81	12	7
Aztreonam *	30	85	6	9
Tobramycin *	10	78	10	12
Tetracycline *	30	74	17	9
Moxifloxacin *	5	76	9	15
Cefoxitin *	30	76	4	20
Ertapenem *	10	83	4	13
Tigecycline *	30	2	0	98
Vancomycin **	0.25–256 µg/mL	14	30	56

* Determined by disc diffusion method; ** Determined by broth microdilution method; n: number of isolates. All isolates were declared resistant, intermediate, or sensitive according to breakpoints provided by Clinical Laboratory Standards Institute.

**Table 2:** Specimen wise distribution of VRSA among MRSA.

Phenotype	Distribution of SA, MRSA, and VRSA from Different Type of Specimens				Chi-Square Test
	Blood *n* (%)	Pus *n* (%)	Sputum *n* (%)	Body Fluid *n* (%)	
SA	25	36	24	15	*p* > 0.05 *
MRSA	19 (76)	29 (80.55)	19 (79.16)	9 (60)	
MRSA^+^ + VRSA^+^	12 (63.16)	14 (48.28)	13 (68.42)	5 (55.56)	
MRSA^+^ + VRSA^−^	7 (36.84)	15 (51.72)	6 (31.58)	4 (44.44)	

MRSA: Methicillin resistant *Staphylococcus aureus*; VRSA: Vancomycin resistant *Staphylococcus aureus*; n: number of isolates; * Significance of Association, *p*-value < 0.05 = significant.

**Table 3:** Background detail on Patient History.

Background	Status	No of Individual
Education of Patients	Illiterate	12
	Less than Matric	48
	Matric to Graduation	40
Economic Status in terms of Income Per month (PKR)	<25,000	53
	25,000–50,000	38
	>50,000	09
Knowledge about Antibiotics	Yes	11
	No	89
Use of Medication	Yes	34
	No	66
Use of Antibiotics for suggested number of days	Yes	05
	No	95

**Table 4:** Antibiotic Susceptibility pattern of methicillin-resistant *Staphylococcus aureus* (MRSA) (*n* = 76).

Antibiotic	MRSA Isolates Showing Susceptibility to Other Antibiotics		
	Resistant *n* (%)	Intermediate *n* (%)	Sensitive *n* (%)
Methicillin *	76 (100)	-	-
Ampicillin *	76 (100)	-	-
Ertapenem *	76 (100)	-	-
Tobramycin *	72 (94.7)	1 (1.3)	3 (3.9)
Tetracycline *	70 (92.1)	3 (3.9)	3 (3.9)
Cefoxitin *	76 (100)	-	-
Moxifloxacin *	68 (89.4)	4 (5.26)	3 (3.9)
Vancomycin **	14 (18.42)	-	32 (42.1)
Tigecycline *	1 (1.3)	-	75 (98.7)

MRSA: Methicillin resistant *Staphylococcus aureus*; *n*: number of isolates; * Determined by disc diffusion method; ** Determined by broth microdilution method. All isolates were declared resistant, intermediate, or sensitive according to breakpoints provided by Clinical Laboratory Standards Institute.

**Table 5:** Antibiotic susceptibility pattern of VRSA (*n* = 44).

Antibiotics	No. of Isolates Showing Antibiotic Susceptibility *		
	Resistant *n* (%)	Intermediate *n* (%)	Sensitive *n* (%)
Methicillin	44 (100)	-	-
Ampicillin	44 (100)	-	-
Cefoxitin	44 (100)	-	-
Tobramycin	43 (97.7)	1 (2.3)	-
Tetracycline	42 (95.5)	1 (2.3)	1 (2.3)
Ertapenem	42 (95.5)	2 (4.5)	-
Moxifloxacin	41 (93.2)	1 (2.3)	2 (4.5)
Tigecycline	1 (2.3)	-	43 (97.7)

* Determined by disc diffusion method and the isolates were declared resistant, intermediate or sensitive according to breakpoints provided by CLSI.

**Table 6:** Primers used in this study.

Target	Nucleotide Sequence of Primers in this Study			Reference
	Primer	Sequence (5′------3′)	Product Size (bp)	
*mec*A	Forward	AGAAGATGGTATGTGGAAGTTAG	583	[34]
	Reverse	ATGTATGTGCGATTGTATTGC		
*van*A	Forward	GGCAAGTCAGGTGAAGATG	713	[11]
	Reverse	ATCAAGCGGTCAATCAGTTC		
