# Correction: Saeed, A., et al. Incidence of Vancomycin-Resistant Phenotype of the Methicillin-Resistant *Staphylococcus aureus* Isolated from a Tertiary Care Hospital in Lahore. *Antibiotics* 2020, 9, 3

**DOI:** 10.3390/antibiotics9020082

**Published:** 2020-02-13

**Authors:** Aqib Saeed, Fatima Ahsan, Muhammad Nawaz, Khadeja Iqbal, Kashif Ur Rehman, Tayyaba Ijaz

**Affiliations:** 1 Department of Microbiology, University of Veterinary and Animal Sciences, Lahore 54000, Pakistan; aqibs114@gmail.com (A.S.); fatima.ahsan@uvas.edu.pk (F.A.); 2 Department of Microbiology, Central Diagnostic Lab, King Edward Medical University/Mayo Hospital Lahore, Lahore 54000, Pakistan; drkhadeja@yahoo.com; 3 Department of Pathology, Central Diagnostic Lab, King Edward Medical University/Mayo Hospital Lahore, Lahore 54000, Pakistan; rehmankashif1004@gmail.com (K.U.R.);

The authors wish to make the following corrections to this paper [1]. The authors would like to apologize for any inconvenience caused to the readers by these changes.

**Change in Main Body Paragraphs**: The authors report that some of the data regarding the minimum inhibitory concentration (MIC) for vancomycin reported in their published paper [1] need a few corrections. Upon revising the experiments following reviewers’ comments, the data were reprocessed. Some of the discrepancies in the reporting of vancomycin-resistant isolates were noted and are corrected here. The MICs of vancomycin for 44 reported *S. aureus* (VRSA) isolates showed that 14 isolates had MICs >16 µg/mL, while the other 30 isolates had MICs in the range of 2–4 µg/mL, revealing that these were vancomycin-intermediate *Staphylococcus aureus* (VISA). The VRSA mentioned in the text and tables in the paper [1] should therefore be read as VRSA/VISA instead of simply VRSA. Consequently, the authors wish to make, at this time, the following corrections to the paper. None of these revisions affect the overall conclusions of the paper [1].

**Abstract:***Staphylococcus aureus* (*S. aureus*)-associated infections are one of the major threats to public health. The aim of the present study was to determine the antibiotic resistance pattern, as well as the genetic characterization, of methicillin and vancomycin-resistant *S. aureus* (VRSA) isolated from a tertiary care hospital in Lahore. The *S. aureus* isolates were isolated from different clinical samples, identified by biochemical testing, and subjected to antibiotic susceptibility testing via the disc diffusion method or broth microdilution method. The methicillin resistance gene (*mec*A) and vancomycin resistance gene (*van*A) were amplified by the polymerase chain reaction. The *S. aureus* isolates showed high incidences of resistance against methicillin (76%) and moderate incidences of resistance to vancomycin (14%). Isolates were also resistant to several other drugs, such as cefoxitin (76%), ertapenem (83%), ampicillin (81%), tobramycin (78%), moxifloxacin (76%), and tetracycline (74%). An encouraging finding was that 98% of isolates were susceptible to tigecycline, indicating its possible role in the treatment of methicillin-resistant *Staphylococcus aureus* (MRSA) and VRSA, as well as the multi-drug resistant *S. aureus*. The *mec*A gene was detected in 33.3% of tested isolates (10/30), while the *van*A gene was also detected in 30% (9/30) of the tested isolates. In conclusion, the frequent presence of methicillin and vancomycin resistance in *S. aureus* appraises the cautious use of these antibiotics in clinical practices. Furthermore, it is suggested that there should be continuous monitoring of tigecycline treatments in clinical setups in order to delay the development of resistance against it.

## 2. Results

The overall result of the current study revealed that clinical isolates of *S. aureus* (*n* = 100) had a high incidence of resistance against methicillin (76%), ampicillin (81%), tobramycin (78%), tetracycline (74%), aztreonam (85%), cefoxitin (76%), and ertapenem (83%), and a moderate occurrence of resistance against vancomycin (14%). The isolates that were resistant to cefoxitin were declared as MRSA [14]. Most of the *S. aureus* isolates (98%) were susceptible to tigecycline (Table 1).

### 2.1. Antibiotic Susceptibility Pattern of MRSA

Among the 100 isolates of *S. aureus*, 76 were resistant to methicillin, all of which (100%) were also resistant to ampicillin and ertapenem (Table 4). MRSA isolates were also found to be resistant to tobramycin (72, 94.7%), tetracycline (70, 92.1%), cefoxitin (76, 100%) and moxifloxacin (68, 89.4%). MRSA isolates also showed co-resistance with vancomycin (14, 18.42%). Out of 76 MRSA isolates, only one (1.3%) isolate was resistant to tigecycline.

### 2.2. Antibiotic Susceptibility Pattern of VRSA/VISA Isolated from MRSA

The antibiotic susceptibility pattern of the 44 VRSA/VISA isolates is presented in Table 5. The vancomycin-resistant phenotype was confirmed by determining the minimum inhibitory concentration (MIC) of isolates against vancomycin via the broth micro-dilution method, as described by the European Committee for Antimicrobial Susceptibility Testing (EUCAST) of the European Society of Clinical Microbiology and Infectious Diseases (ESCMID) and the Clinical Laboratory Standards Institute (CLSI) [15,16]. The MICs of vancomycin for 44 reported VRSA/VISA showed that 14 isolates had MICs > 16 µg/mL while the other 30 isolates had MICs in the range of 2–4 µg/mL, revealing that these were vancomycin-intermediate *S. aureus* (VISA). The data also showed that most of the VRSA/VISA isolates were also resistant to ampicillin (44/44, 100%), cefoxitin (44/44, 100%), tobramycin (43/44), tetracycline (42/44), and moxifloxacin (41/44). Only one VRSA/VISA isolate was found resistant to tigecycline (2.2%) (see Table 5).

### 2.3. Amplification and Partial Sequencing of mecA and vanA

Out of 30 isolates, the vancomycin-resistant gene *van*A was amplified from nine (30%) isolates (Figure 2).

Additional Note: Like the replacement shown in Section 2.2, the “VRSA” mentioned in the text and tables elsewhere in paper [1] should also be read as “VRSA/VISA” instead of “VRSA”.

## Figures and Tables

**Table 1:** Antibiotic sensitivity pattern of *Staphylococcus aureus* (*n* = 100).

Antibiotic	Quantity (µg)	Antibiotic Sensitivity Pattern of *S. aureus* (*n* = 100)		
		Resistant	Intermediate	Sensitive
Methicillin *	5	76	11	13
Ampicillin *	10	81	12	7
Aztreonam *	30	85	6	9
Tobramycin *	10	78	10	12
Tetracycline *	30	74	17	9
Moxifloxacin *	5	76	9	15
Cefoxitin *	30	76	4	20
Ertapenem *	10	83	4	13
Tigecycline *	30	2	0	98
Vancomycin **	0.25–256 µg/mL	14	30	56

* Determined by disc diffusion method; ** Determined by broth microdilution method; *n*: number of isolates. All isolates were declared resistant, intermediate, or sensitive according to breakpoints provided by Clinical Laboratory Standards Institute.

**Table 4:** Antibiotic Susceptibility pattern of methicillin-resistant *Staphylococcus aureus* (MRSA) (*n* = 76).

Antibiotic	MRSA Isolates Showing Susceptibility to Other Antibiotics		
	Resistant *n* (%)	Intermediate *n* (%)	Sensitive *n* (%)
Methicillin *	76 (100)	−	−
Ampicillin *	76 (100)	−	−
Ertapenem *	76 (100)	−	−
Tobramycin *	72 (94.7)	1 (1.3)	3 (3.9)
Tetracycline *	70 (92.1)	3 (3.9)	3 (3.9)
Cefoxitin *	76 (100)	−	−
Moxifloxacin *	68 (89.4)	4 (5.26)	3 (3.9)
Vancomycin **	14 (18.42)	30 (39.47)	32 (42.1)
Tigecycline *	1 (1.3)	−	75 (98.7)

Methicillin-resistant *Staphylococcus aureus* (MRSA); *n*: number of isolates; * Determined by disc diffusion method; ** Determined by broth microdilution method. All isolates were declared resistant, intermediate, or sensitive according to breakpoints provided by the Clinical Laboratory Standards Institute.
